# Inhibition of alveolar Na transport and LPS causes hypoxemia and pulmonary arterial vasoconstriction in ventilated rats

**DOI:** 10.14814/phy2.12985

**Published:** 2016-09-26

**Authors:** Bodo Davieds, Julian Gross, Marc M. Berger, Emel Baloğlu, Peter Bärtsch, Heimo Mairbäurl

**Affiliations:** ^1^ Medical Clinic VIISports MedicineUniversity of HeidelbergHeidelbergGermany; ^2^ Department of AnesthesiologyPerioperative and General Critical Care MedicineSalzburg General HospitalParacelsus Medical UniversitySalzburgAustria; ^3^ Translational Lung Research Center Heidelberg (TLRC‐H)Member of the German Center for Lung Research (DZL)HeidelbergGermany; ^4^ Department of PharmacologyAcibadem UniversityIstanbulTurkey

**Keywords:** Alveolar reabsorption, amiloride, hypoxia, inflammation, pulmonary edema, pulmonary vascular resistance

## Abstract

Oxygen diffusion across the alveolar wall is compromised by low alveolar oxygen but also by pulmonary edema, and leads to hypoxemia and hypoxic pulmonary vasoconstriction (HPV). To test, whether inhibition of alveolar fluid reabsorption results in an increased pulmonary arterial pressure and whether this effect enhances HPV, we established a model, where anesthetized rats were ventilated with normoxic (21% O_2_) and hypoxic (13.5% O_2_) gas received aerosolized amiloride and lipopolisaccharide (LPS) to inhibit alveolar fluid reabsorption. Right ventricular systolic pressure (RVsP) was measured as an indicator of pulmonary arterial pressure. Oxygen pressure (PaO_2_) and saturation (SaO_2_) in femoral arterial blood served as indicator of oxygen diffusion across the alveolar wall. Aerosolized amiloride and bacterial LPS decreased PaO_2_ and SaO_2_ and increased RVsP even when animals were ventilated with normoxic gas. Ventilation with hypoxic gas decreased PaO_2_ by 35 mmHg and increased RVsP by 10 mmHg. However, combining hypoxia with amiloride and LPS did not aggravate the decrease in PaO_2_ and SaO_2_ and had no effect on the increase in RVsP relative to hypoxia alone. There was a direct relation between SaO_2_ and PaO_2_ and the RVsP under all experimental conditions. Two hours but not 1 h exposure to aerosolized amiloride and LPS in normoxia as well as hypoxia increased the lung wet‐to‐dry‐weight ratio indicating edema formation. Together these findings indicate that inhibition of alveolar reabsorption causes pulmonary edema, impairs oxygen diffusion across the alveolar wall, and leads to an increased pulmonary arterial pressure.

## Introduction

Decreased alveolar oxygen pressure (PO_2_) causes hypoxic pulmonary vasoconstriction (HPV), and its degree varies with the severity of hypoxia. The oxygen sensor triggering HPV seems to be located in the smooth muscle cells (for review see (Sylvester et al. 2012)). Mainly alveolar but also systemic PO_2_ controls this response (Marshall and Marshall 1983). HPV occurs not only when inspired PO_2_ is low such as at high altitude (Hultgren et al. 1964) but also in situations such as pneumonia (Zapol and Snider 1977) and adult respiratory distress syndrome (ARDS) (Ryan et al. 2014), where oxygen diffusion across the alveolar wall is impaired by edema causing hypoxemia and pulmonary arterial vasoconstriction (Price et al. 2012). This stresses the significant role of pulmonary edema in increasing pulmonary arterial pressure (Albert and Jobe 2012). Pulmonary edema is a consequence of augmented filtration due to elevated hydrostatic pressure in pulmonary capillaries and increased alveolar permeability; it can also be caused by impaired reabsorption of alveolar fluid (for review see (Matthay and Ingbar 1998)).

Alveolar reabsorption removes excess fluid from the alveolar surface in order to optimize the diffusion distance for respiratory gasses across the alveolar wall. Consequently, an intact fluid clearance correlated with improved arterial oxygenation and clinical outcome in ARDS patients (Verghese et al. 1999). Hypoxia increases fluid filtration due to an increase in pulmonary capillary pressure (Parker et al. 1979), which plays a role, for example, in high altitude pulmonary edema (HAPE) (Maggiorini et al. 2001). Hypoxia also inhibits alveolar water reabsorption by decreasing active Na reabsorption (e.g., Baloğlu et al. (2011); Güney et al. (2007); Vivona et al. (2001)). The resultant accumulation of fluid in the alveolar space further impaired oxygen diffusion and thus may contribute to the magnitude of HPV. In fact, individuals who develop HAPE have exaggerated HPV and also have decreased Na‐ and fluid reabsorption in the lung indicated by the surrogate of lower nasal potential differences than healthy controls (Sartori et al. 2002; Mairbäurl et al. 2003; Betz et al. 2015). However, a direct relation between alveolar reabsorption and pulmonary vasoconstriction has not been demonstrated.

It was the aim of this study to set up a model where it can be tested whether inhibition of alveolar reabsorption impairs alveolar oxygen diffusion and increases pulmonary arterial systolic pressure even at normal alveolar PO_2_, and whether inhibited reabsorption aggravates physiological HPV. In this model anesthetized and ventilated rats received aerosolized amiloride or bacterial endotoxin lipopolisaccharide (LPS) to inhibit apical alveolar epithelial Na channels and thus fluid reabsorption. Our results indicate that these treatments in fact cause hypoxemia in rats ventilated with normoxic gas and increase right ventricular systolic pressure (RVsP). However, we could not demonstrate that inhibition of alveolar reabsorption in hypoxic animals augments the physiologic HPV.

## Methods

Male Wistar rats, weight ~300 g (Janvier, Le Genest, France), were anesthetized by i.p. injection of 90 mg/kg S‐ketamine (Ketanest©, Pfizer, Dun Laoghaire, Ireland) and 15 mg/kg xylazine (Rompun©, Bayer, Leverkusen, Germany) and were heparinized (250 U). The animals were kept on a heating plate to maintain normal body temperature. A tracheal tube was inserted for mechanical ventilation (Hugo Sachs Electronics, March‐Hugstetten, Germany) with a tidal volume of 6 mL/kg at 80 per min and a positive end‐expiratory pressure (PEEP) of 2 cm H_2_O; the inspiration‐to‐expiration ratio was 1:1.5. Every 15 min PEEP was increased from 3 to 10 cm H_2_O for 10 sec to prevent atelectasis. This protocol was chosen to minimize ventilator‐induced lung injury and to maintain a constant alveolar PO_2_ at the respective oxygenation level to standardize the driving force for oxygen diffusion across the alveolar barrier.

A catheter filled with saline was inserted into the jugular vein and was advanced into the right ventricle to record RVsP as a surrogate for pulmonary arterial systolic pressure. Proper placement of the catheter was monitored with the pressure curves. Another saline filled catheter was inserted into the femoral artery to record systemic systolic blood pressure (FAsP). Pressure transducers were connected to amplifiers (DBA; Hugo Sachs Electronics). The output signal was digitized using PowerLab (AdInstruments, Speckbach, Germany) and recorded continuously on a PC. The PowerLab software detects pressure peaks and frequency to obtain systolic values from the pressure curves and the heart rate, respectively.

After instrumentation rats were allowed to stabilize for 15 min while being ventilated with normoxic room air indicated by stable values of FAsP and RVsP. Rats were then disconnected from the respirator for ~1 min for intratracheal application of saline (0.9% NaCl) (100 *μ*L), amiloride (100 *μ*L of 0.9 mmol/L amiloride), or LPS (100 *μ*L of 55 *μ*g/mL) using a microsprayer (Penn‐Century, Wyndmoor, PA). This amount of amiloride caused an approximately 50% inhibition of alveolar fluid clearance (independent experiments, not shown) measured by fluid instillation as described earlier (Baloğlu et al. 2011). Mechanical ventilation was then continued with room air (normoxia) or with a hypoxic gas mixture composed of 13.5% O_2_ and 86.5% N_2_. Femoral arterial blood samples (80 *μ*L) were collected for blood gas analyses at the end of the equilibration period and every 15 min thereafter.

Experiments were terminated by removing the lung after 60 or 120 min. For the measurement of the wet‐weight‐to‐dry‐weight ratio, an indicator of pulmonary edema, lungs were cleaned from adhering blood, and trachea and main bronchi were removed. The tissue was weighed before and after drying for 48 h at 80°C. The protocol was approved by the Animal Protection Committee of the University of Heidelberg and by the Regierungspräsidium Karlsruhe, Germany.

### Data evaluation

**Figure 1:**
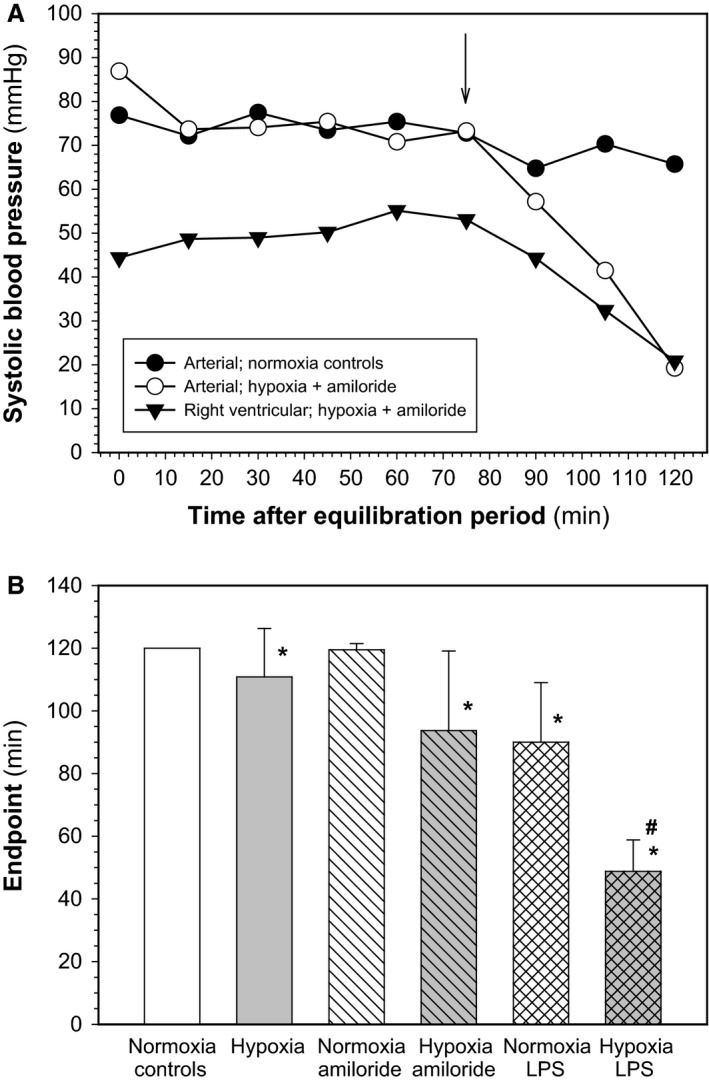
(A) Study end points in 2 h experiments. During the 2 h experiments a nonrecoverable decrease in systolic femoral arterial and a concomitant decrease in right ventricular pressure occurred indicating hemodynamic instability of anesthetized, ventilated rats in some experimental conditions. The time of its occurrence was defined as “endpoint” (indicated by the arrow). As an example, representative curves of FAsP and RVsP from animals exposed to hypoxia plus amiloride are shown in comparison to FAsP from a normoxic control rat. (B) Mean values ± SD of “endpoints” defined as indicated in (A) for the different experimental conditions (number of experiments: normoxia control 18; hypoxia 16; normoxia + amiloride 15; hypoxia + amiloride 22; normoxia + lipopolisaccharide (LPS) 4; hypoxia + LPS 11). *Significant effect of hypoxia; ^#^significant effect of hypoxia in LPS‐treated animals.

We performed two series of experiments, where the experiments were terminated after 60 and 120 min after the equilibration period, respectively. We had to add 60 min lasting experiments because in some experimental conditions FAsP and RVsP begun to decrease after prolonged exposure. Figure 1A shows examples indicating that FAsP was stable over the entire 2 h observation period in normoxic control rats that received aerosolized saline. However, in rats ventilated with hypoxic gas and that also received aerosolized amiloride, FAsP begun to decrease after ~ 80 min, that is, well before the planned 2 h end point. At the same time also RVsP begun to decrease, likely because of decreased venous return. Therefore, we defined as study “endpoint” the time after equilibration, when a nonrecoverable drop in FAsP >15 mmHg begun, which we determined retrospectively. This end point varied significantly between experimental conditions (Fig. 1B). Thus, in the 2 h experiments wet and dry weights were obtained from lungs collected after completing the 2‐h exposure, whereas PaO_2_, SaO_2_, FAsP, and RVsP reported are from the respective end point. Conditions were stable in the 1 h series.

The values for RVsP, FAsP, and HR shown in the figures are mean values from the last 5 min before the indicated time ‐points, that is, the end of the equilibration phase (co), 60 min, and the end point. Blood gas data shown are from the end of the equilibration period, 60 min, and the time point closest to the end point. Results are shown as mean values ± SD. One‐way ANOVA was used to calculate changes over time within each experimental condition and to compare values at the respective end points from different experimental conditions, followed by Student‐Newman‐Keul post hoc testing for group comparisons. Statistics were calculated using the SigmaPlot^®^ and SigmaStat^®^ software package (Systat Inc., Erkrath, Germany). A *P* < 0.05 was considered statistically significant.

## Results

Figure 1B shows mean values of end points for all studied experimental conditions as defined in Figure 1A. All normoxic control animals that received aerosolized saline were stable over the full 2 h observation period (end point = 2 h), whereas the end point was slightly earlier in hypoxic animals that received aerosolized saline (*P* = 0.001). In animals treated with aerosolized amiloride in normoxia the end point was not different from normoxic controls (*P* = 0.782), but it was decreased significantly when amiloride was combined with hypoxia (*P* = 0.001). Alveolar application of LPS significantly decreased the end point in normoxia (*P* = 0.005) and in hypoxia (*P* = 0.001). Treatment with aerosolized amiloride and LPS in hypoxia significantly decreased the end point relative to hypoxia alone (*P* = 0.001).

**Figure 2:**
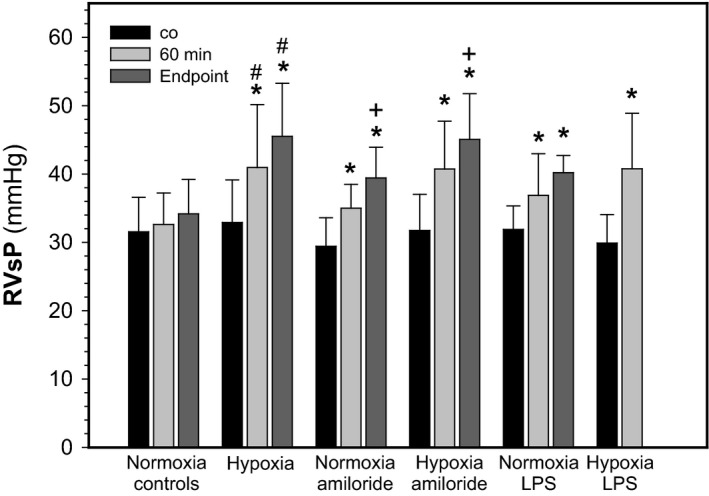
Effects of hypoxia, amiloride, and lipopolisaccharide on right ventricular systolic pressure. RVsP at the end of the equilibration phase (co), and after 60 min (*n* = 11–27 per experimental condition) and at the end point (*n* see legend to Fig. 1). Mean values ± SD. *P < 0.05 relative to co; ^+^ *P* < 0.05 between 60 min and the end point; # indicated difference between normoxia and hypoxia in the respective experimental condition.

Figure 2 shows that RVsP was about 32 mmHg at the end of the equilibration period, that is, before treatments begun (co), which is in the range of values reported in the literature (e.g., McMurtry et al. (1978)). There was no significant difference between groups at this time point (*P* = 0.249). The figure also shows that aerosolized saline did not significantly affect RVsP in normoxia during the 60 and 120 min observation periods (*P* = 0.162). In contrast, hypoxia significantly increased RVsP by 8 mmHg after 60 min (*P* < 0.001), and by 13.5 mmHg at the end point (*P* = 0.001); there was only a tendency toward statistical significance between the two time points (*P* = 0.082). Aerosolized amiloride in normoxic animals significantly increased RVsP by 5.5 mmHg after 60 min (*P* = 0.001) and by 10 mmHg at the end point (*P* = 0.001; 60 min vs. endpoint: *P* = 0.002). Amiloride in combination with hypoxia significantly increased RVsP by 9 mmHg after 60 min (*P* = 0.001) and by 13.3 mmHg by the end point (*P* = 0.001; 60 min vs. end point: *P* = 0.02). Aerosolized LPS in normoxia increased RVsP by ~5 mmHg (*P* = 0.012) after 60 min and by 8.4 mmHg at the end point (*P* = 0.005); there was no significant difference between the time points (*P* = 0.233). Hypoxia plus LPS increased RVsP 11 mmHg (*P* = 0.001). There is no later time point available because of circulatory instability in the longer experiments. Neither amiloride (*P* = 0.847) nor LPS (*P* = 0.954) applied in hypoxia increased RVsP more than hypoxia alone indicating lack of additivity of effects.

Femoral arterial systolic blood pressure was ~95 mmHg at the end of the equilibration period (Table 1A; *P* = 0.194). There was no change in FAsP in normoxic animals treated with saline (*P* = 0.412), with amiloride (*P* = 0.134), as well as in hypoxia (*P* = 0.081), but a statistically significant decrease in FAsP in hypoxia plus amiloride (*P* = 0.005), and in normoxia plus LPS (*P* = 0.05) and hypoxia plus LPS (*P* = 0.016). Heart rates (Table 1B) were ~230 per min after the equilibration period, which is within the reported range (Flindt 1995). Heart rates were not changed by experimental conditions in animals ventilated with normoxic gas (*P* > 0.40), but were increased significantly in all experiments under hypoxic conditions (*P* < 0.05).

**Table 1:** Effects of hypoxia, amiloride, and lipopolisaccharide (LPS) on femoral arterial systolic pressure (FAsP) and heart rates

	co	60 min	Endpoint
(A) FAsP (mmHg)			
normoxia + saline	90.3 ± 18.3	85.8 ± 25.0	83.8 ± 18.4
hypoxia + saline	86.0 ± 16.0	79.7 ± 20. 8	77.8 ± 12.9
normoxia + amiloride	85.0 ± 10.0	82.9 ± 10.9	80.1 ± 17.9
hypoxia + amiloride	93.1 ± 18.5	74.2 ± 16.7	79.1 ± 15.0^a^
normoxia + LPS	102.3 ± 25.8	79.4 ± 15.3	78.6 ± 12.9^a^
hypoxia + LPS	88.8 ± 13.3	74.7 ± 12.0^a^	
(B) Heart rates (per min)			
normoxia + saline	224 ± 52	228 ± 50	231 ± 37
hypoxia + saline	213 ± 51	252 ± 45^a^	249 ± 28^a^
normoxia + amiloride	221 ± 57	230 ± 51	219 ± 47
hypoxia + amiloride	220 ± 43	246 ± 45^a^	232 ± 45
normoxia + LPS	233 ± 40	245 ± 39	218 ± 24
hypoxia + LPS	203 ± 70	255 ± 35^a^	

Data are from the end of the equilibration phase (co), and after 60 min (*n* = 11–27 per experimental condition) and at the end point of treatment (*n* see legend to Fig. 1). Mean values ± SD.

a Indicate *P* < 0.05 relative to co.

**Figure 3:**
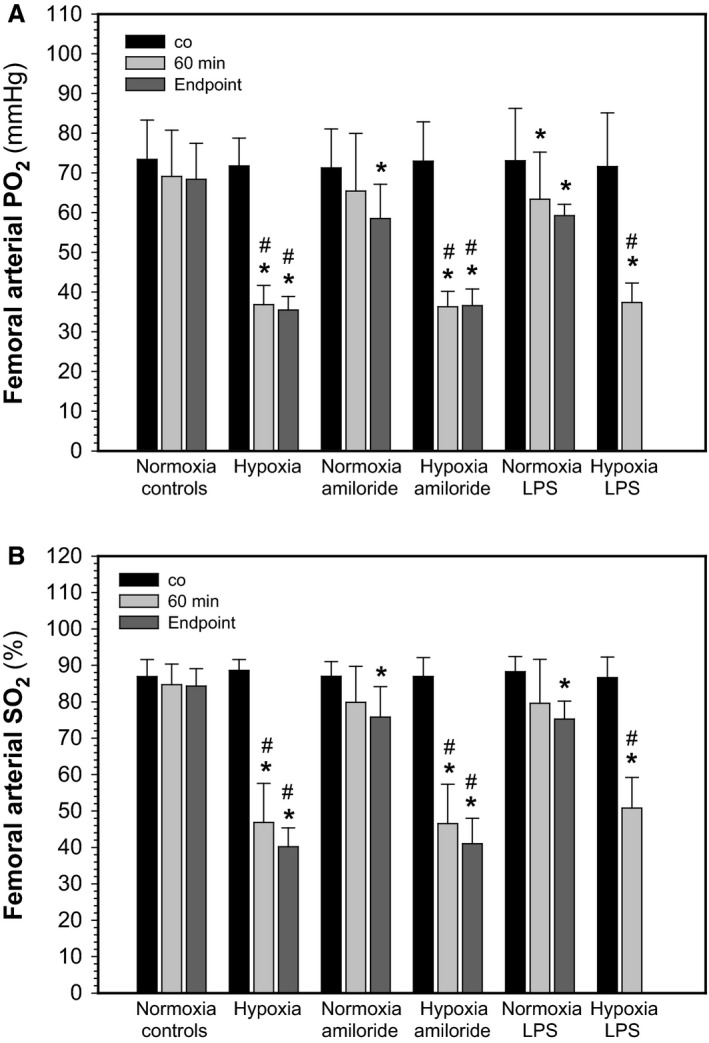
Effects of hypoxia, amiloride, and lipopolisaccharide on femoral arterial oxygen partial pressure (PO_2_; A) and oxygen saturation (SO_2_: B). Data from the end of the equilibration phase (co), and after 60 min (*n* = 11–27 per experimental condition) and at the end point of treatment (*n* see legend to Fig. 1). Mean values ± SD. *P < 0.05 relative to co; ^#^ *P* < 0.05 between normoxia and hypoxia at the respective condition.

Any impairment of alveolar function that causes alveolar and/or interstitial edema should also compromise oxygen diffusion across the alveolar barrier. Figure 3A and B shows that at the end of the equilibration period PaO_2_ and SaO_2_ were ~73 mmHg and ~88%, respectively. In normoxic controls PaO_2_ (*P* = 0.082) and SaO_2_ (*P* = 0.091) had decreased slightly in saline‐treated animal after 2 h. Ventilating rats with a hypoxic gas (13.5% O_2_) significantly decreased PaO_2_ by 35 mmHg and SaO_2_ by about 40% (*P* < 0.001). There was no statistically significant difference between 60 min and the end point. In normoxia, amiloride aerosolization caused a small decrease in PaO_2_ (*P* = 0.05) and SaO_2_ (*P* = 0.005) at the end point. There was no significant difference between 60 min and the end point. Amiloride in hypoxia decreased PaO_2_ by 36 mmHg and SaO_2_ by 41% (*P* = 0.001). LPS in normoxia decreased PaO_2_ (*P* = 0.028) and SaO_2_ (*P* = 0.05), and also in hypoxia (PaO_2_ −36 mmHg, *P* < 0.001; SaO_2_ −37%, *P* < 0.001). Neither amiloride nor LPS in combination with hypoxia decreased PO_2_ and SO_2_ more than hypoxia alone indicating lack of additivity of these effects.

**Figure 4:**
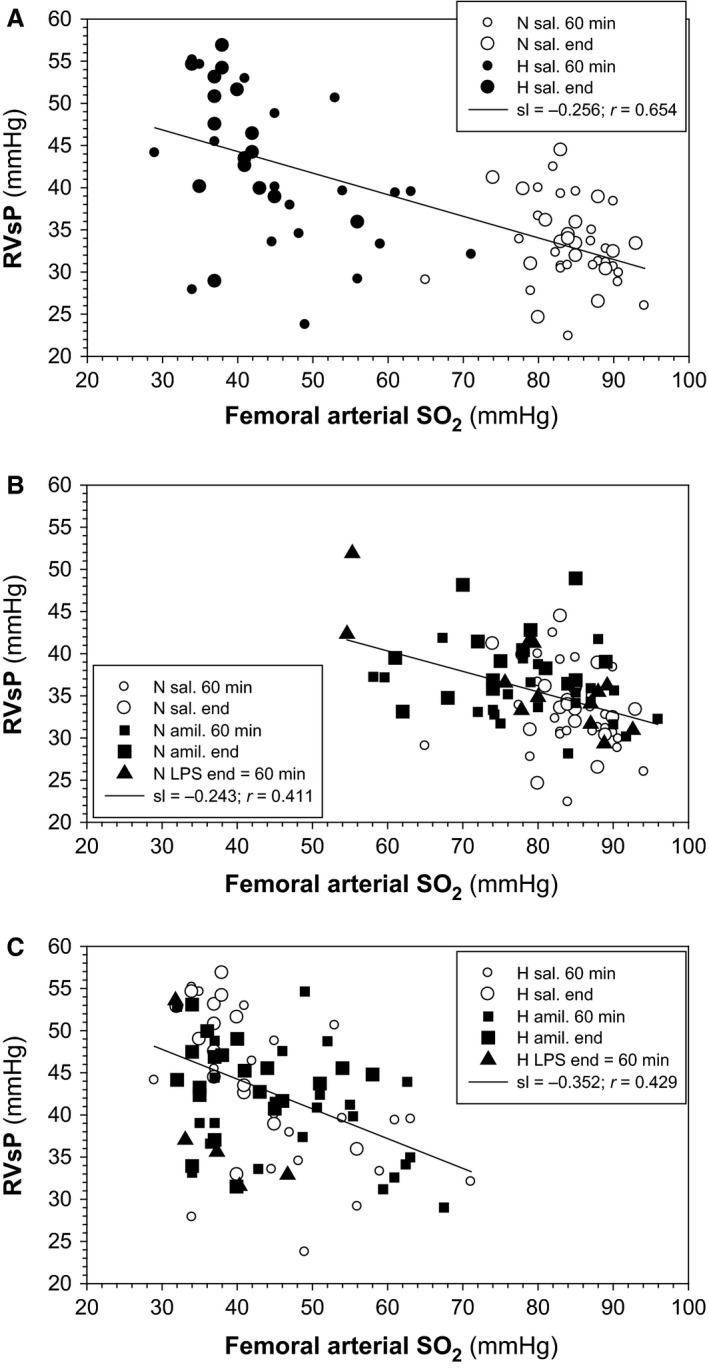
Inverse correlations between right ventricular systolic pressure (RVsP) and femoral arterial SO_2_. Data from experiments with (A) normoxia‐saline and hypoxia‐saline, (B) normoxia‐saline, normoxia‐amiloride, and normoxia‐lipopolisaccharide (LPS), and (C) hypoxia‐saline, hypoxia‐amiloride, and hypoxia‐LPS. N, normoxia; H, hypoxia; sal., aerosolized saline; sl, slope of the regression line (mmHg per % SO_2_).

Because the degree of HPV has been shown to depend on the degree of hypoxia (Sylvester et al. 2012), we correlated PaO_2_ and SaO_2_ in femoral arterial blood with RVsP to test whether this was also true in our experimental setting. Figure 4 shows inverse correlations between SaO_2_ and RVsP. Regression coefficients are shown in the inserts. Figure 4A shows that RVsP increased significantly with decreasing SaO_2_ (*R* = 0.654; *P* < 0.001) when plotting results from normoxic and hypoxic animals that received aerosolized saline. The slope (sl) of the regression line amounted to 0.252 mmHg per 1% decrease in SaO_2_. In comparison, Figure 4B shows that significant correlations existed also for SaO_2_ and RVsP, when animals were ventilated with normoxic gas and were treated with saline, amiloride, and LPS (*R* = 0.411, *P* = 0.001). Plotting only the data from hypoxic animals (treatments saline, amiloride, or LPS, 60 min and end points) also resulted in a statistically significant correlation (Fig. 4C; *R* = 0.429; *P* = 0.001). Similar correlations were found between RVsP and PaO_2_ (not shown).

**Figure 5:**
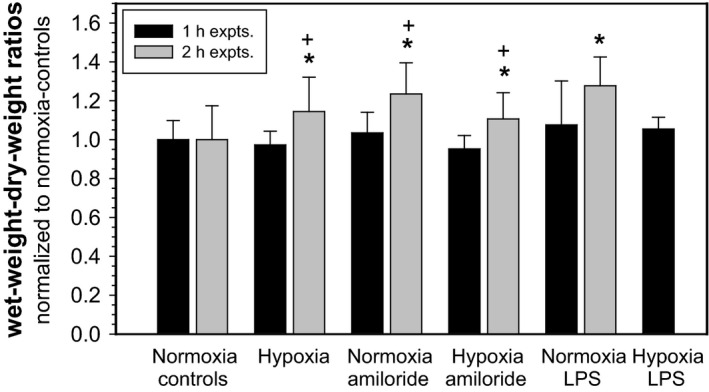
Effects of hypoxia, amiloride, and lipopolisaccharide (LPS) on the wet‐to‐dry‐weight ratio of rat lungs. In one series experiments were terminated after 1 h (*n* = 7–10 per experimental condition), in a second 2 h after equilibration (*n* = 4 [normoxia + LPS] to 22) for collecting lung tissue samples to determine the wet‐to‐dry‐weight ratios. Mean values ± SD; *P < 0.05 relative to normoxia control; ^+^ *P* < 0.05 between 1 and 2 h.

Wet‐to‐dry‐weight ratios were measured to see whether inhibition of alveolar reabsorption and hypoxia caused pulmonary edema. Figure 5 shows wet‐weight‐to‐dry‐weight ratios from the 1 and 2 h experiments, normalized to mean values from normoxic controls. It has to be noted that the latter values were obtained from lungs after 2 h and not at the defined endpoint (see Fig. 1A and B). There was no difference in the wet‐to‐dry‐weight ratios between experimental conditions in the 1 h experiments (*P* = 0.292). In contrast, 2 h after the treatment lung water was significantly increased in hypoxic animals that received aerosolized saline (*P* = 0.026), in normoxic animals receiving amiloride (*P* = 0.001), hypoxia plus amiloride (*P* = 0.044), and normoxia plus LPS (*P* = 0.010). There was a significant difference in the wet‐to‐dry ratios between 1 and 2 h experiments in hypoxia (*P* = 0.035), in hypoxia plus amiloride (*P* = 0.008), and in normoxia with LPS (*P* = 0.003).

## Discussion

Hypoxia causes pulmonary arterial vasoconstriction (Sylvester et al. 2012). Hypoxia of pulmonary vascular smooth muscle cells is caused by impaired oxygen diffusion across the alveolar wall as a consequence of decreased alveolar PO_2_ and a thickening of the alveolar barrier by interstitial and alveolar edema. Because the latter can also be caused by impaired alveolar fluid clearance (Matthay et al. 2002), we tested whether inhibition of alveolar reabsorption affects pulmonary arterial pressure, independent of alveolar PO_2_. Indeed, we found that amiloride and LPS increased right ventricular systolic pressure in rats ventilated with normoxic gas, similar to hypoxic pulmonary arterial vasoconstriction.

We used a model, where anesthetized rats were ventilated with normoxic or hypoxic gas with a constant tidal volume and respiratory rate to maintain a constant alveolar PO_2_ at the respective oxygen content of the gas used for ventilation. Aerosolized amiloride and LPS were applied to inhibit the reabsorption of alveolar fluid in order to increase the volume of alveolar lining fluid, which should impair oxygen diffusion across the alveolar wall indicated by a decrease in femoral arterial PO_2_ and SO_2_. We considered changes in arterial oxygenation to be a more sensitive parameter than lung water content measured as the wet‐to‐dry‐weight ratio to indicate alveolar edema because we found the latter unchanged even though PO_2_ and SO_2_ were already decreased. This is in line with findings indicating that already very small changes in lung water, which are difficult to detect as weight change, are sufficient to impair oxygen diffusion (Tschumperlin and Margulies 1999; Ochs 2006).

The volume of alveolar lining fluid depends strongly on the activity of alveolar Na transport. Amiloride and LPS are well‐known inhibitors of epithelial Na transport (Eaton et al. 2009). Both affect mainly epithelial Na channels (ENaC) located at the alveolar surface, which, together with basolateral Na/K‐ATPase, mediate vectorial Na transport and thus generate the osmotic driving force required for the removal of water from the alveolar surface (Eaton et al. 2009; Matalon et al. 2015). The significance of alveolar reabsorption is best documented by the fact that impaired alveolar fluid clearance correlates with bad clinical outcome in ARDS patients (Matthay and Wiener‐Kronish 1991; Ware and Matthay 2001). Consequently, a decreased in the activity of ENaC should result in increased lung water and hypoxemia by impaired oxygen diffusion. In fact we show here that both, aerosolized amiloride and LPS, decrease femoral arterial PO_2_ and SO_2_ even when animals were ventilated with normoxic gas. As postulated, this resulted in a significant increase in RVsP.

Alveolar Na‐ and water reabsorption is inhibited by hypoxia. This has been demonstrated in cultured primary alveolar epithelial cells (e.g., Mairbäurl et al. (2002); Planes et al. (2002)) as well as in rats exposed to hypoxia (e.g., Baloğlu et al. (2011); Güney et al. (2007); Vivona et al. (2001)). Furthermore, the lung water content of hypoxic rats was increased (e.g., Stelzner et al. (1988)), which we confirm here. The increase in lung water seems to occur slowly because we did not see it after 1 h but only after 2 h of ventilating rats with hypoxic gas. This indicates that alveolar hypoxia can lead to pulmonary vasoconstriction by two means: one is vasoconstriction due to the decreased alveolar PO_2_; a second is an increased alveolar water content, which may be a consequence of increased filtration due to a hypoxia‐induced increase in pulmonary capillary pressure (Maggiorini et al. 2001), which is not adequately removed due to hypoxic inhibition of alveolar reabsorption. Both effects might add and even enhance water accumulation as well as the resultant hypoxemia, and thus, also of HPV. Our experiments do not allow quantifying the contribution of each of the two effects.

Correlation analysis revealed a statistically significant increase in RVsP with arterial deoxygenation. Hypoxemia caused by ventilation with a hypoxic gas resulted in an increase in RVsP by ~0.25 mmHg per percent SO_2_. This response is approximately 50% of that estimated from data reported by Marshall and Marshall (1983) and McMurtry et al. (1978) for rats, and from other species (Table 1 in Sylvester et al. (2012)). The difference may be due to differences in the experimental settings. Interestingly, we found that in normoxic animals RVsP increased by a similar magnitude, when hypoxemia was caused by inhibiting alveolar reabsorption with amiloride or LPS (Fig. 4). Although correlation provides no basis for a causal relationship, this result indicates that the increase in RVsP by hypoxia of pulmonary vascular smooth muscle cells depends not only on alveolar PO_2_ alone but also on factors that limit the rate of oxygen diffusion across the alveolar barrier.

Based on these findings we expected that combining ventilation with a hypoxic gas and inhibition of alveolar reabsorption might impair oxygen diffusion and cause an increase in RVsP, respectively, that was more pronounced than by each intervention alone, resulting in an exaggerated increase in RVsP. However, our results do not support this hypothesis. Neither femoral arterial PO_2_ decreased more after inhibition of alveolar reabsorption in hypoxic animals than with hypoxia alone, nor was there a more pronounced increase in RVsP.

We can only speculate on reasons that might explain the lack of additivity. One might be the shorter time to reach the end point in hypoxic animals that also received aerosolized amiloride or LPS because of a shorter time for alveolar fluid accumulation and therefore less impairment of O_2_ diffusion. Another reason might be diffusion of amiloride to vascular smooth muscle cells. Amiloride inhibits Ca entry into smooth muscle cells, which weakens contraction (Cribbs 2006; Kuo et al. 2011), and systemically applied amiloride has been shown to blunt HPV by Ca‐dependent mechanisms (Raffestin and McMurtry 1987). In fact, significant plasma levels have been found in humans after inhaling amiloride (Jones et al. 1997). An increased alveolar permeability and protein leakage into the alveolar space in hypoxic rats (Stelzner et al. 1988) enhance amiloride diffusion. Thus, diffusion of amiloride may have prevented an exaggerated increase in RVsP resulting in reduced filtration of water into the alveolar space, and thus a smaller effect on oxygen diffusion. Another factor might be a reduction in the number of Na channels in hypoxic alveolar epithelium (Planes et al. 2002) because it reduces the degree of inhibition of Na transport by amiloride. Together these effects might prevent the additivity of effects of breathing hypoxic gas and alveolar transport inhibition on arterial hypoxemia and RVsP.

A major limitation of our model is the limited stability of circulation, which seems to occur upon a drop in arterial PO_2_ below approximately 35 mmHg and an oxygen content of inspiratory air below 13.5% as tested in preliminary experiments (not shown). This phenomenon is likely caused by anesthesia and constant ventilation, which prevents circulatory and ventilatory adjustments to hypoxia. By contrast, awake rats tolerate normobaric hypoxia (inspiratory O_2_ 8%) very well (e.g., Baloğlu et al. (2011)). The higher degree of hypoxemia upon combining ventilation with a hypoxic gas and inhibition of alveolar reabsorption was therefore likely the reason for the earlier onset the circulatory instability and may have prevented seeing additivity of effects.

## Conclusion

Our results show that inhibition of alveolar fluid reabsorption by inhibition of Na transport with amiloride and LPS impairs oxygen diffusion, likely by alveolar edema. This results in hypoxemia, and causes a similar degree of increase in pulmonary arterial pressure as ventilating rats with hypoxic gas.

## Conflicts of Interest

There are no conflicts of interest to be declared.
